# Host Factors That Interact with the Pestivirus N-Terminal Protease, N^pro^, Are Components of the Ribonucleoprotein Complex

**DOI:** 10.1128/JVI.00984-14

**Published:** 2014-09

**Authors:** Matthew Jefferson, Andras Donaszi-Ivanov, Sean Pollen, Tamas Dalmay, Gerhard Saalbach, Penny P. Powell

**Affiliations:** a Biomedical Research Centre, Norwich Medical School, University of East Anglia, Norwich, Norfolk, United Kingdom; b Biological Sciences, University of East Anglia, Norwich, United Kingdom; c John Innes Centre, Norwich Research Park, Colney, Norwich, United Kingdom

## Abstract

The viral N-terminal protease N^pro^ of pestiviruses counteracts cellular antiviral defenses through inhibition of IRF3. Here we used mass spectrometry to identify a new role for N^pro^ through its interaction with over 55 associated proteins, mainly ribosomal proteins and ribonucleoproteins, including RNA helicase A (DHX9), Y-box binding protein (YBX1), DDX3, DDX5, eIF3, IGF2BP1, multiple myeloma tumor protein 2, interleukin enhancer binding factor 3 (IEBP3), guanine nucleotide binding protein 3, and polyadenylate-binding protein 1 (PABP-1). These are components of the translation machinery, ribonucleoprotein particles (RNPs), and stress granules. Significantly, we found that stress granule formation was inhibited in MDBK cells infected with a noncytopathic bovine viral diarrhea virus (BVDV) strain, Kyle. However, ribonucleoproteins binding to N^pro^ did not inhibit these proteins from aggregating into stress granules. N^pro^ interacted with YBX1 though its TRASH domain, since the mutant C112R protein with an inactive TRASH domain no longer redistributed to stress granules. Interestingly, RNA helicase A and La autoantigen relocated from a nuclear location to form cytoplasmic granules with N^pro^. To address a proviral role for N^pro^ in RNP granules, we investigated whether N^pro^ affected RNA interference (RNAi), since interacting proteins are involved in RISC function during RNA silencing. Using glyceraldehyde-3-phosphate dehydrogenase (GAPDH) silencing with small interfering RNAs (siRNAs) followed by Northern blotting of GAPDH, expression of N^pro^ had no effect on RNAi silencing activity, contrasting with other viral suppressors of interferon. We propose that N^pro^ is involved with virus RNA translation in the cytoplasm for virus particle production, and when translation is inhibited following stress, it redistributes to the replication complex.

**IMPORTANCE** Although the pestivirus N-terminal protease, N^pro^, has been shown to have an important role in degrading IRF3 to prevent apoptosis and interferon production during infection, the function of this unique viral protease in the pestivirus life cycle remains to be elucidated. We used proteomic mass spectrometry to identify novel interacting proteins and have shown that N^pro^ is present in ribosomal and ribonucleoprotein particles (RNPs), indicating a translational role in virus particle production. The virus itself can prevent stress granule assembly from these complexes, but this inhibition is not due to N^pro^. A proviral role to subvert RNA silencing through binding of these host RNP proteins was not identified for this viral suppressor of interferon.

## INTRODUCTION

Pestiviruses are a group of small positive-stranded RNA viruses in the Flavivirus family that cause economically important diseases of farm animals and include bovine viral diarrhea virus (BVDV), classical swine fever virus (CSFV), and border disease virus (BDV) (1). The genome, of approximately 12 kb, encodes only 12 proteins, which are co- and posttranslationally processed from a single RNA into the N-terminal protease (N^pro^), capsid, E1, E2, p7, NS2, NS3, NS4A, NS4B, NS5A, and NS5B (2). Pestiviruses replicate in the cytoplasm with no nuclear component and mature in intracellular vesicles that are thought to arise from the endoplasmic reticulum (ER) and are released by exocytosis (3). Translation is initiated by an internal ribosome entry site (IRES), which binds the 40S ribosomal subunit and eIF3 to direct cap-independent translation, but translation does not require the translation initiation factors eIF4B and eIF4F (4). The N-terminal protease, N^pro^, is a 168-amino-acid autoprotease, unique to pestiviruses, with cysteine protease activity in a Glu22-His49-Cys69 triad that acts to cleave itself from the nascent polypeptide (5). Deletion of N^pro^ has no effect on viral replication or virulence (6); however, interestingly, expression of N^pro^ alone in cells has been shown to suppress the innate immune response by inhibiting apoptosis and interferon production (7, 8).

The innate immune response to infection takes place through cellular recognition of double-stranded RNA replication intermediates by a family of RNA helicases (9, 10), including protein kinase R, which phosphorylates initiation factor eIF2 to arrest translation and promotes stress granule formation (11), RNA helicase A, which can pair with MAVS/IPS-1 (12) and also activate NF-κB (13), and importantly, RIG-I and MDA-5, which transduce the signal for interferon production (14, 15). Most if not all viruses encode proteins that block these innate responses to double-stranded RNA (dsRNA) (16). In the case of pestiviruses, we have previously found that in infected cells, IRF3, a central regulator of interferon transcription and initiator of apoptosis, is lost, which inhibits dsRNA-induced interferon alpha and beta expression (7, 17). The loss of IRF3 is due to a single viral protein, N^pro^, and direct interaction of N^pro^ with IRF3 has been demonstrated by coimmunoprecipitation (17), although it is not known whether this involves recruitment of other cellular proteins. Unique to pestiviruses, the structure of N^pro^ has been solved to 1.25-Å resolution (18) and contains two compact units, a protease domain and an interaction domain. The interaction domain contains a novel metal-binding TRASH motif at the C-terminal end, consisting of Cys-X21-Cys-X3-Cys at its C terminus, which coordinates a single zinc atom, and mutations at this site attenuate its properties. TRASH domains are abundant in prokaryotes but are also found in a few vertebrate proteins (19).

To learn more about the function of N^pro^, in this study we have taken a proteomic approach to investigate cellular proteins that interact with it, using mass spectrometry and pull-down analysis. We found that this viral protein interacts directly with 40S and 60S ribosomal proteins and initiation factor eIF3a, dependent on the presence of zinc. N^pro^ also bound to several RNA helicases and dsRNA binding proteins, including RNA helicase A (DHX9), YBX1, DDX3X, and DDX5. In fact, many of the interacting cellular proteins are components of cytoplasmic ribonucleoprotein particles (RNPs), responsible for controlling mRNA translation and which can be assembled into stress granules to control the rate of translational initiation or mRNA decay (20). Stress granule formation may also regulate virus replication, and some viruses may inhibit the formation of or disassemble these granules during infection (21). To investigate whether the interaction of N^pro^ with ribonucleoproteins influences their function and localization, we examined whether cells expressing N^pro^ or infected with BVDV and then subjected to oxidative stress were protected from stress granule formation. BVDV-infected cells did not form stress granules when exposed to oxidative stress; however, this was not due to N^pro^ binding to ribonucleoproteins, since cells expressing N^pro^ alone formed stress granules and N^pro^ codistributed with stress granule proteins. The RISC complex is an important component of RNPs, and we asked if N^pro^ had a functional role in inhibiting RNA interference, as has been shown for other viral inhibitors of interferon synthesis (22, 23).

## MATERIALS AND METHODS

### Reagents and virus infection.

HEK-293 (human embryonic kidney) cells were maintained in Dulbecco's modified Eagle medium (DMEM)-Glutamax (Invitrogen) supplemented with 10% heat-inactivated fetal calf serum. N^pro^-mCherry was generated from pcDNA3 N^pro^ (7) and cloning in frame into pcDNA3mCherry (Invitrogen). N^pro^-mCherry C112R and D136N point mutants were created using a QuikChange kit according to the manufacturer's instructions (Stratagene). mCherry-tagged TIA1, pEGFP-tagged RNA helicase A (RHA), V5-tagged YBX-1 V5, DDX1-V5, and DCP1a, and Myc-tagged DDX3 were variously transfected into cells with Fugene HD (Roche). Transfected cells were allowed 24 h to recover at 37°C before stimulation with 100 μM sodium arsenate (NaA). Plasmids used were obtained from the following sources: pDDX3-myc was from Andrew Bowie and Martina Schoder (24), pEGFP- RHA full length (amino acids [aa] 1 to 1270) and pEGFP-RHA R1163A with a mutation at aa 1163 were a gift from T. Nakajima, Department of Genome Science, Institute of Medical Science, St. Marianna University School of Medicine, Kanagawa, Japan (25). pDDX1-V5, pDCP1a-V5, and YBX-V5 were from S. Ishura, University of Tokyo (26). The mCherry-tagged construct TIA1 was from T. Eisinger-Mathason, University of Virginia, as described in reference 27. La-V5 was generated by T. Csorba (University of East Anglia [UEA], Norwich, United Kingdom). N^pro^ antibody raised to peptide KTNKQKPMGVEEPVYDATGKPLFGDPS, which corresponds to amino acids 11 to 37 of the Alfort strain of CSFV, linked to keyhold limpet hemocyanin (KLH) and ovalbumin (OVA), was raised in rabbits as described in reference 7. Mouse anti-RHA antibody (ab54593) and rabbit anti-YBX1 (ab12148) were from Abcam. Alpha-actin antibodies were from Sigma. For virus infections, bovine MDBK cells were grown in minimal essential medium (MEM) and 10% BVDV-free medium with Glutamax (Invitrogen). Cells were seeded on coverslips in 24-well plates and infected with the BVDV Kyle noncytopathic strain of BVDV (from J. Brownlie, Royal Vet College, South Mimms, United Kingdom). After absorption of virus for 1 h, cells were fed with fresh medium and incubated overnight at 37°C before treatment or transfection with DNA.

### GST Pulldown analysis.

Bacteria (Escherichia coli BL21) were transformed with plasmids encoding glutathione *S*-transferase (GST) or GST-N^pro^, cultured for 4 h, and then induced with 100 mM isopropyl-b-d-thiogalactopyranoside (IPTG) for 4 h. Bacteria were lysed with the Bug Buster Protein extraction reagent (Merck). Lysates were clarified by centrifugation, resuspended in low-salt pulldown buffer (LPD) (20 mM Tris-HCl [pH 7.6], 200 mM NaCl, 0.5% NP-40, 0.5 mM dithiothreitol [DTT], and 0.4 mM phenylmethylsulfonyl fluoride [PMSF]) and 1 mg/ml protease inhibitors, and added to 3 ml of 50% glutathione–Sepharose bead slurry (Amersham Biosciences) overnight at 4°C. Beads were washed 7 times in a high-salt pulldown buffer (HPD) (500 mM NaCl and 1% Triton) to remove nonspecific proteins and reequilibrated with LPD with or without 1 mM zinc chloride to obtain beads binding either GST alone or GST N^pro^.

HEK 293 cell were lysed in MPER buffer (Pierce), and supernatants were clarified by centrifugation. Lysates were precleared by adding glutathione beads overnight at 4°C, and beads were separated by centrifugation. Precleared supernatants were added to GST-Sepharose beads or GST-N^pro^-Sepharose beads as appropriate. Beads were washed seven times in LPD buffer, and proteins were analyzed by SDS-PAGE. For mass spectrometry analysis, proteins were eluted from the beads with 10 mM or 30 mM reduced glutathione.

For V5 antibody pulldowns, HEK 293 cells were transfected with plasmids expressing protein with a V5 tag for 24 to 48 h, and cells were lysed in MPER with 1 mM ZnCl and incubated on ice for 30 min. In some experiments, a zinc chelator, N,N,N′,N′-Tetrakis (2-pyridylmethyl)ethylenediamine (TPEN), was used. Lysates were precleared with 50% slurry protein G-Sepharose for 1 h, before addition of anti-V5 antibody followed by protein G Sepharose beads. Beads were washed seven times in in LPD, resuspened in sample preparation buffer, and analyzed by SDS-PAGE followed by Western blotting.

### Identification of virus-host protein partners by mass spectrometry.

Proteins were eluted from washed beads by adding 2-fold-concentrated SDS-gel sample loading buffer and heating to 80°C for 10 min. The supernatant was loaded onto a Novex gel (10% Bis-Tris SDS gel; Life Technologies/Invitrogen, Carlsbad, CA) and run about 1/3 of the length of the lane. The lanes were cut out and stained with InstantBlue (Expedeon Ltd., Harston, United Kingdom) in separate trays until bands were just visible. The area covering the bands was cut into 5 to 6 slices, which were washed, reduced, and alkylated and treated with trypsin according to standard procedures. Peptides were extracted with 5% formic acid–50% acetonitrile, dried down, and redissolved in 0.1% trifluoroacetic acid (TFA). For liquid chromatography-tandem mass spectrometry (LC-MS/MS) analysis, a sample aliquot was applied via a nanoAcquity (Waters, Manchester, United Kingdom) ultraperformance liquid chromatography (UPLC) system running at a flow rate of 250 nl min^−1^ to an LTQ-Orbitrap mass spectrometer (Thermo Fisher, Waltham, MA). Peptides were trapped using a precolumn (Symmetry C_18_, 5 μm, 180 μm by 20 mm; Waters), which was then switched in-line to an analytical column (BEH C_18_, 1.7 μm, 75 μm by 250 mm; Waters) for separation. Peptides were eluted with a gradient of 3 to 40% acetonitrile in water–0.1% formic acid at a rate of 0.67% min^−1^. The column was connected to a 10-μm SilicaTip nanospray emitter (New Objective, Woburn, MA, USA) attached to a nanospray interface (Proxeon, Odense, Denmark) for infusion into the mass spectrometer. The mass spectrometer was operated in positive ion mode at a capillary temperature of 200°C. The source voltage and focusing voltages were tuned for the transmission of Met-Arg-Phe-Ala (MRFA) peptide (*m*/*z* 524) (Sigma-Aldrich, St. Louis, MO). Data-dependent analysis was carried out in orbitrap-ion trap parallel mode using collision-induced fragmentation (CID) on the 6 most abundant ions in each cycle. The orbitrap was run with a resolution of 30,000 over the MS range from *m*/*z* 350 to *m*/*z* 1,800 and an MS target of 10^6^ and 1-s maximum scan time. Collision energy was 35, and an isolation width of 2 was used. Only monoisotopic 2+ and 3+ charged precursors were selected for MS2 fragmentation stage. The MS2 was triggered by a minimal signal of 1,000 with an automatic gain control target of 3 × 10^4^ ions and 150-ms scan time using the chromatography function for peak apex detection. Dynamic exclusion was set to 1 count and 60-s exclusion with an exclusion mass window of ±20 ppm. MS scans were saved in profile mode, while MS/MS scans were saved in centroid mode.

Raw files were processed using the software program MaxQuant, version 1.3.0.5 (28) (http://maxquant.org), to generate recalibrated peak list files, which were used for a database search using an in-house Mascot 2.4 server (Matrix Science Limited, London, United Kingdom). Mascot mgf files were generated from MaxQuant apl files using a suitable Perl script. Mascot searches were performed on the Sprot_sptrembl20121031.fasta database with taxonomy set to human using trypsin/P with 2 missed cleavages, 6-ppm precursor tolerance, 0.6-Da fragment tolerance, carbamidomethylation (C) as fixed, and oxidation (M) and acetylation (protein N terminus) as variable modifications. Mascot search results were imported and evaluated in the program Scaffold 3.6.1 (Proteome Software, Portland, OR, USA), resulting in a false-discovery rate of 0% for both peptides and proteins. For the detection of GST and CSFV N^pro^, the protein sequences of those proteins were added to a custom database with 1,000 random E. coli sequences (downloaded from uniprot.org) as a background, and the searches were performed in the same way as described above.

The mass spectrometry proteomics data have been deposited in the ProteomeXchange Consortium (http://proteomecentral.proteomexchange.org) via the PRIDE partner repository (29) with the data set identifier PXD000115 and doi number 10.6019/PXD000115 (PRIDE accession numbers 28015 to 28026).

### Immunoblotting.

Proteins were separated by SDS-PAGE (10 to 15% acrylamide) and transferred to polyvinylidene difluoride (PVDF) membranes (0.45-um transfer membrane; Thermo Scientific). Membranes were blocked with 5% (wt/vol) dried skimmed milk in Tris-buffered saline (TBS) containing 0.5% Tween 20. Membranes were probed with antibodies to YBX, V5, N^pro^, and RNA helicase A. Proteins were detected with IRDye-labeled secondary antibodies (926-322 and 926-680; Li-Cor Biosciences) at a 1:10,000 dilution or with horseradish peroxidase (HRP)-conjugated antibodies (111-035-003; Jackson Laboratory). Proteins detected by the labeled secondary antibodies were visualized on the Odyssey infrared system or enhanced chemiluminescence detection of horseradish peroxidase activity.

### Immunocytochemistry and fluorescence microscopy.

HEK293 cells were blocked in goat serum gelatin quench and permeabilized with 0.2% Triton X-100. Primary antibodies were diluted into 0.2% Triton X-100 in goat gelatin quench and incubated with cells for 1 h at room temperature, and cells were washed three times in 0.1% Tween. Antibodies used were anti-RHA monoclonal antibody (MAb) (ab54593; Abcam), anti-V5 MAb (R960; Invitrogen), anti-Myc 9E10 MAb, rabbit anti-YB1 (ab12148; Abcam), and anti-ubiquitin FK2 MAb (BML-PW8810; Enzo).

### Caspase 3/7 apoptosis assay.

Following cell treatment, caspase activity was measured in cell lysates using the caspase 3/7 Glo assay (Promega) in 96-well plates with the addition of the luminescent substrate Z-DEVD. Luciferase activity was measured using an Envision plate reader (PerkinElmer). Fold induction of caspase activity was obtained by dividing the activity in the treated samples by the activity obtained in the untreated cells for either uninfected cells or cells infected with BVDV.

### GAPDH silencing and Northern blot analysis.

Glyceraldehyde-3-phosphate dehydrogenase (GAPDH)- or RHA-specific small interfering RNA (siRNA) (Dharmacon) at a concentration of 50 mM was transfected into cells for 24 h using the JetPrime reagent (Polyplus). Total RNA was isolated (GeneElute; Sigma), separated on formaldehyde gels, and blotted onto nitrocellulose membranes. The membrane was hybridized with ^32^P-labeled primer against GAPDH (GGCATGGACTGTGGTCATGAG) and 18S RNA (TTACAGGGCCTCGAAAGAGT).

## RESULTS

### Host proteins interacting with N^pro^ are components of ribosomes and ribonucleoprotein particles.

Orbitrap mass spectrometry was used to identify novel cellular proteins interacting with N^pro^ from the classical swine fever virus strain Alfort. Purified recombinant GST-N^pro^ protein was incubated with HEK 293 cell lysates that had either been untreated or transfected with synthetic dsRNA, poly(I·C), for 4 h both to induce expression of early-response genes that may be important following viral infection and to investigate the requirement of dsRNA for proteins to bind to N^pro^. Table 1 shows 45 proteins that were specifically eluted from N^pro^-GST compared to results with GST alone, with either a low (10 mM) or high (30 mM) glutathione concentration and with or without poly(I·C). The list is in order of the number of peptides found for each protein. (A full list is given in the PRIDE partner repository, with the data set identifier PXD000095 and doi number 10.6019/PXD000095 (PRIDE accession no. 28008). Interestingly, many of the hits were ribosomal proteins and dsRNA binding proteins, which form ribonucleoprotein particles in the cytoplasm and which are involved in RNA metabolism. Transfection of dsRNA poly(I·C) into cells for 4 h before lysis made very little difference in the proteins detected, indicating that binding to N^pro^ was not dependent on dsRNA, normally present in cells only during viral infection and replication. In addition to ribosomal proteins, interacting proteins included Y-box binding protein (YBX-1), La autoantigen, RNA helicase A (DHX9), multiple myeloma tumor protein 2, interleukin enhancer binding factor 3 (IEBP3), guanine nucleotide binding protein 3, and polyadenylate binding protein 1 (PABP-1). These proteins are involved in RNA metabolism, including translation and mRNA degradation, and they are normally found distributed through the cytoplasm of cells (30). It may be that N^pro^ interacts with one or two of these RNA binding proteins, and the other proteins are pulled down in the same complex. Surprisingly, we found no direct binding of N^pro^ to the immune modulators IRF3, IRF7, Hax-1, or I-κBα, which have previously been described by yeast 2-hybrid interaction (17, 31–34) in cells of immune lineage. This may reflect differences between yeast 2-hybrid and protein pulldown mass spectrometry, that expression levels of these proteins are low in HEK293 cells compared to those in immune cells, or the fact that additional proteins are required. Our previous work has shown that N^pro^ is able to bind and degrade IRF3 in HEK 293 cells (8).

**TABLE 1:** Cellular proteins associated with GST-tagged N^pro*a*^

Peptide abundance rank	Accession no.	Protein identified	Mol mass^*b*^ (kDa)	Association with N^pro^ at GSH concn (mM) of:							
				10				30			
				No pI·C		pI·C		No pI·C		pI·C	
				% ID^*c*^	No. of peptides	% ID	No. of peptides	% ID	No. of peptides	% ID	No. of peptides
1	P09211	Glutathione *S*-transferase	23	100	7	100	10	100	9	100	9
2	P61247	40S ribosomal protein S3a	30	100	6	100	4	100	3	100	4
3	P15880	40S ribosomal protein S2	31	100	3	100	1	100	3	100	3
4	Q00839	Heterogeneous nuclear ribonucleoprotein U	91	100	6	100	2	100	4	100	4
5	P67809	Y-box-binding protein 1 (YBX-1)	36	100	4	100	4	100	5	100	4
6	P62424	60S ribosomal protein L7a	30	100	3	85	1	100	3	100	5
7	P19338	Nucleolin	77	100	4	100	2	100	3	100	4
8	P62750	60S ribosomal protein L23a	18	100	4	100	4	100	2	100	3
9	P39023	60S ribosomal protein L3	46	100	3	99	1	100	2	83	1
10	P62263	40S ribosomal protein S14	16	100	2	100	1	100	2	100	2
11	P05455	La autoantigen, Sjoegren syndrome type B antigen (SS-B)	47	100	3	100	2	100	3	100	3
12	Q5JR94	40S ribosomal protein S8	24	100	2	99	1	100	1	100	3
13	Q59FI9	Ribosomal protein L12	21	100	2	100	2	100	2	100	2
14	P26373	60S ribosomal protein L13	24	100	2	99	1	100	1	100	3
15	P62244	40S ribosomal protein S15a	15	100	2	100	2	100	2	99	1
16	P46782	40S ribosomal protein S5	23	100	2	100	2	100	2	100	3
17	P62277	40S ribosomal protein S13	17	100	3	100	2	100	2	99	1
18	Q59ES8	Heterogeneous nuclear ribonucleoprotein M	65	100	3	93	0	100	3	100	2
19	P62081	40S ribosomal protein S7	22	100	1	46	0	100	1	99	1
20	P83731	60S ribosomal protein L24	18	100	2			99	0	100	3
21	Q08211	ATP-dependent RNA helicase A (RHA, DEAH box protein 9)	141	100	2			100	4	83	1
22	P18621	60S ribosomal protein L17	21	100	2	100	2	100	2	100	2
23	P61254	60S ribosomal protein L26	17	100	1	98	1	99	0	100	1
24	Q53HK9	Ribosomal protein P0	34	100	1	85	1	100	1	100	2
25	P18124	60S ribosomal protein L7	?	85	1	99	1	99	1	100	2
26	P62899	60S ribosomal protein L31	14	100	2	100	2	100	2	100	2
27	P39019	40S ribosomal protein S19	16			100	2	87	1	100	2
28	P38159	Heterogeneous nuclear ribonucleoprotein G (hnRNP G)	42	100	2			100	2	83	1
29	Q9BU76	Multiple myeloma tumor-associated protein 2	29	100	2	85	1	99	1	100	2
30	Q12906	Interleukin enhancer-binding factor 3 (TCP80)	95	100	2			100	2	100	2
31	P84098	60S ribosomal protein L19	23	100	2			100	1	60	0
32	P83881	60S ribosomal protein L36a	12	98	1	84	0	100	1	83	1
33	P61313	60S ribosomal protein L15	24	98	1	33	0	87	1	99	1
34	P36578	60S ribosomal protein L4	48	85	1			99	1	100	1
35	P31943	Heterogeneous nuclear ribonucleoprotein H (hnRNP H)	49	100	3	85	1	36	0	48	0
36	Q9BVP2	Guanine nucleotide-binding protein-like 3	62	100	1	85	1	100	2		
37	P61978	Heterogeneous nuclear ribonucleoprotein K (hnRNP K)	51	85	1			100	2	100	2
38	Q8NC51	Plasminogen activator inhibitor 1 RNA-binding protein	45	85	1	85	1			100	2
39	P08579	U2 small nuclear ribonucleoprotein B″ (SU2 snRNP B)	25	99	1			100	2	70	0
40	Q9Y3D9	28S ribosomal protein S23 (mitochondrial)	22	100	1	85	1	48	0		
41	P11940	Polyadenylate-binding protein 1 (PABP-1)	71	99	1					100	2
42	P27635	60S ribosomal protein L10	25					100	2	83	1
43	O60832	H/ACA ribonucleoprotein complex subunit 4	58	60	0			99	1		
44	P22626	Heterogeneous nuclear ribonucleoproteins A2/B1	37	56	0			99	1		
45	Q59GL1	Synaptotagmin binding, cytoplasmic RNA interacting protein variant	60					100	1		

a N^pro^ interacts with ribosomal and ribonucleoproteins, components of ribonuclear particles (RNPs). Interaction of N^pro^ with cellular proteins was identified by mass spectrometry analysis. Recombinant GST-tagged N^pro^ was used to pull down cellular binding partners from HEK293 cells. Cells were either control (“no. pI·C”) or transfected with poly(I·C) (pI·C) (dsRNA) for 4 h before lysis. Beads were eluted with low (10 mM) or high (30 mM) glutathione (GSH). Proteins binding to GST alone were used for a control. The accession number and molecular mass for each protein are shown in order of abundance of peptides found.

b Molecular mass.

c ID, identity.

N^pro^ is a metalloprotein coordinating a single zinc ion through a putative metal binding domain called a TRASH motif at Cys112-X21-Cys134-X3-Cys138 (19). This domain is thought to be required for IRF3 interaction and degradation (34). In order to investigate which proteins were interacting with the zinc binding TRASH domain in the C-terminal end of N^pro^, in a second experiment, the recombinant protein was bound to lysates either in the presence of zinc or in the presence of a chelator of zinc, TPEN. The interacting proteins are shown in Table 2 (deposited in PRIDE, accession no. 28009). In this experiment, a total of 55 cellular proteins were found to bind N^pro^ (Table 2), of which 9 were also detectedin the group shown in Table 1 (numbers in parentheses in column 1 of Table 2). There were 28 additional proteins in the presence of zinc and only 2 extra proteins bound when zinc was absent (desmoglein and desmoplakin); 25 proteins bound irrespective of the presence of zinc. Interestingly, the proteins that required zinc were involved in RNA translation, and included signal recognition particle, RNA helicases DDX 5 and DHX 15, eukaryotic initiation factor eIF3i, and tRNA ligases. These are ribonucleoproteins, also present in polysomes, stress granules, and P bodies. For some proteins that bound N^pro^ both in the presence and absence of zinc, the number of peptides increased in the presence of zinc, showing increased affinity and suggesting some interaction with the TRASH motif in N^pro^. Heterogeneous ribonucleoproteins hnRNP M, U, A/B/C, and H and G were found as cellular binding partners, and these proteins are involved in mRNA nuclear export, localization, translation, and stability, important in both the nucleus and the cytoplasm. In addition, many of these factors are also involved in posttranscriptional gene regulation.

**TABLE 2:** Mass spectrometry identification of cellular proteins binding to N^pro^ with or without zinc^*a*^

Peptide abundance rank^*b*^	Accession no.	Protein identified	Mol mass^*c*^ (kDa)	Binding to N^pro^ with:			
				No Zn		Zn present	
				% binding	No. of peptides	% ID^*d*^	No. of peptides
1 (21)	Q08211	ATP-dependent RNA helicase A; DEAH box protein 9	141	100	30	100	50
2	P28161	Glutathione *S*-transferase	26	100	11	100	9
3	C7DJS2	Glutathione *S*-transferase	17	100	2	100	2
4	P52272	Heterogeneous nuclear ribonucleoprotein M	78	100	6	100	6
5 (2)	P61247	40S ribosomal protein S3a	30	100	1	100	7
6	P23396	40S ribosomal protein S3	27	100	2	100	7
7	P54577	Tyrosine-tRNA ligase	59			100	9
8	B5BTY4	ATP-dependent RNA helicase DDX3X	73	100	2	100	6
9	B1AHM1	DEAD (Asp-Glu-Ala-Asp) box polypeptide 17	73	99	1	100	5
10	Q9NZI8	Insulin-like growth factor 2 mRNA-binding protein 1	63	100	2	100	5
11	P12956	Lupus Ku autoantigen protein p70	70	39	0	100	5
12 (38)	Q8NC51	Plasminogen activator inhibitor 1 RNA-binding protein	45	62	0	100	2
13	P46782	40S ribosomal protein S5	23	78	1	100	5
14	P62701	40S ribosomal protein S4	30	78	1	100	3
15	Q9Y262	Eukaryotic translation initiation factor 3 subunit L (eIF3l)	67	35	0	100	3
16	Q92900	ATP-dependent helicase RENT1	124	99	1	100	3
17 (41)	P11940	Polyadenylate-binding protein 1 (PABP-1)	71	25	0	100	3
18	P61011	Signal recognition particle 54-kDa protein; (SRP54)	56	18	0	100	2
19	O76094	Signal recognition particle 72-kDa protein (SRP72)	75			100	3
20	Q9Y285	Phenylalanine-tRNA ligase alpha subunit	58			100	3
21 (45)	Q59GL1	Synaptotagmin binding, cytoplasmic RNA interacting protein	60			100	3
22	Q0VAC0	Heterogeneous nuclear ribonucleoprotein A1	34	100	2	99	1
23	B3KNR3	Glutathione *S*-transferase	19	99	1	99	1
24 (19)	P62081	40S ribosomal protein S7	22	98	1	100	2
25 (30)	Q12906	Interleukin enhancer-binding factor 3; TCP80	95	78	1	100	3
26	Q13435	Splicing factor 3B subunit 2	100	78	1	100	2
27	Q9UHB9	Signal recognition particle 68-kDa protein	71			100	3
28	P04844	Ribophorin II (RPN-II)	69			100	2
29	P49458	Signal recognition particle 9-kDa protein; SRP9	10			100	2
30	Q9H6T3	RNA polymerase II-associated protein 3	76			100	3
31 (11)	P05455	La autoantigen; Sjoegren syndrome type B antigen (SS-B)	47			100	3
32	Q02543	60S ribosomal protein L18a	21			100	3
33	P37108	Signal recognition particle 14-kDa protein	15			100	2
34	P07910	Heterogeneous nuclear ribonucleoproteins C1/C2	34	78	1	100	2
35	P62913	60S ribosomal protein L11	20	78	1	100	2
36	P62266	40S ribosomal protein S23	16	41	0	100	2
37	P62277	40S ribosomal protein S13	17			100	3
38	Q02413	Desmoglein 1	114	100	3		
39	Q6NVW7	Importin subunit alpha	58			100	2
40	Q9BUJ2	Heterogeneous nuclear ribonucleoprotein U-like protein 1	96			100	2
41	P14923	Desmoplakin 3	82	100	1		
42	P46778	60S ribosomal protein L21	19			100	1
43	Q96EY7	Pentatricopeptide repeat-containing protein 3 (mitochondrial)	79			100	1
44	P26640	Valine-tRNA ligase	140			99	1
45	F8W7C6	60S ribosomal protein L10	19			100	2
46	O43143	ATP-dependent RNA helicase DHX15 (DEAH box protein 15)	91			100	2
47	P17844	ATP-dependent RNA helicase DDX5; DEAD box protein 5	69			100	2
48 (44)	P22626	Heterogeneous nuclear ribonucleoproteins A2/B1	37			100	2
49	Q13347	Eukaryotic translation initiation factor 3 (eIF-3-beta)	37			100	2
50	Q53GX7	Threonyl-tRNA synthetase variant	83			100	2
51	Q99729	Heterogeneous nuclear ribonucleoprotein A/B	36			100	1
52	Q53F35	Acidic (leucine-rich) nuclear phosphoprotein 32	29			100	1
53	P62851	40S ribosomal protein S25	14			99	1
54	Q9Y5A9	YTH domain family protein 2; high-glucose-regulated protein 8	62			99	1
55	P18077	60S ribosomal protein L35a	13			99	1

a N^pro^ binding to cellular proteins through TRASH domain by zinc chelation. Cellular proteins associated with N^pro^ in the presence or absence or zinc, detected by Orbitrap mass spectrometry analysis.

b The accession number and molecular mass for each protein are shown in order of abundance of peptides found. Proteins also identified in Table 1 are indicated by numbers in parentheses.

c Molecular mass.

d ID, identity.

### BVDV infection inhibits stress granule formation.

**FIG 1:**
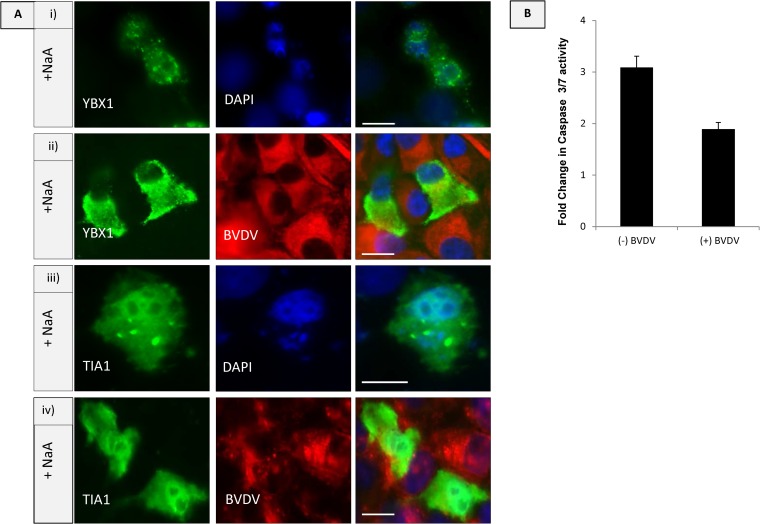
(A) MDBK cells infected with BVDV are protected from stress-induced apoptosis. (i) Control MDBK cells transfected with YBX1-V5 and treated with NaA for 4 h. (ii) Cells infected with BVDV for 48 h postinfection (hpi), transfected with YBX1-V5 overnight, and treated with NaA for 4 h. YBX-V5 was detected with anti-V5 MAb and anti-mouse Alexa 488 secondary antibody. Virus was detected with V182 hyperimmune serum and anti-bovine cy5 secondary antibody. (iii) MDBK cells transfected with TIA1-mCherry plasmid and treated with NaA for 4 h. (TIA was pseudocolored green for consistency). (iv) MDBK cells infected with BVDV for 48 hpi, transfected with TIA1-mCherry, and treated with NaA for 4 h. Virus was detected with V182 hyperimmune serum and anti-bovine cy5 secondary antibody, and TIA1 was pseudocolored green. (B) BVDV infection decreases stress-induced caspase 3/7 activation. MDBK cells, either uninfected (−) or infected with (+) BVDV noncytopathic (ncp) Kyle for 48 h, were stressed with NaA for 4 h. The graph shows the fold increase in caspase activity normalized to that of control cells and significance with one -sided *t* test value, *P* = 0.00004.

Virus infection may trigger stress in cells. The ribonucleoproteins shown to interact with N^pro^ described above are known to aggregate, after exposure of cells to stress, into RNA granules known as stress granules, which regulate initiation, termination, and decay of mRNAs (20). Since several viruses have been shown to actively inhibit stress granule assembly at certain times postinfection, possibly to allow synthesis of their own proteins (35), we asked whether pestivirus infection affects stress granule formation. Arsenate treatment is widely used for oxidative stress to induce stress granule formation (20), and in the next experiments, cells were treated with arsenate in the presence and absence of BVDV (Fig. 1A). TIA1, an RNA binding protein which nucleates stress granule formation, was used as a stress granule marker (36). TIA1 is not only a translational silencer but also a key protein in the prion-like aggregation of further mRNPs to stress granules. YBX1, shown above to interact with N^pro^, is a major component of cellular RNPs which accumulates in granules following cellular stress. MDBK cells expressing YBX1 or TIA1 alone and treated with sodium arsenate for 4 h showed YBX1 and TIA1 accumulation in stress granules, and cells died by apoptosis, seen as DNA fragmentation (Fig. 1Ai and iii). In cells infected with BVDV for 48 h, YBX1 and TIA1 remained cytoplasmic following sodium arsenate treatment for 4 h (Fig. 1Aii and iv), and they did not accumulate in stress granules or in virus replication centers. Furthermore, BVDV-infected cells were protected from apoptosis following sodium arsenate treatment. This experiment is shown using a quantitative caspase 3/7 assay in Fig. 1B, where there is a decrease in caspase activity after sodium arsenate treatment of BVDV-infected cells compared to that for uninfected cells.

### N^pro^ interaction with YBX1 does not prevent formation of stress granules.

**FIG 2:**
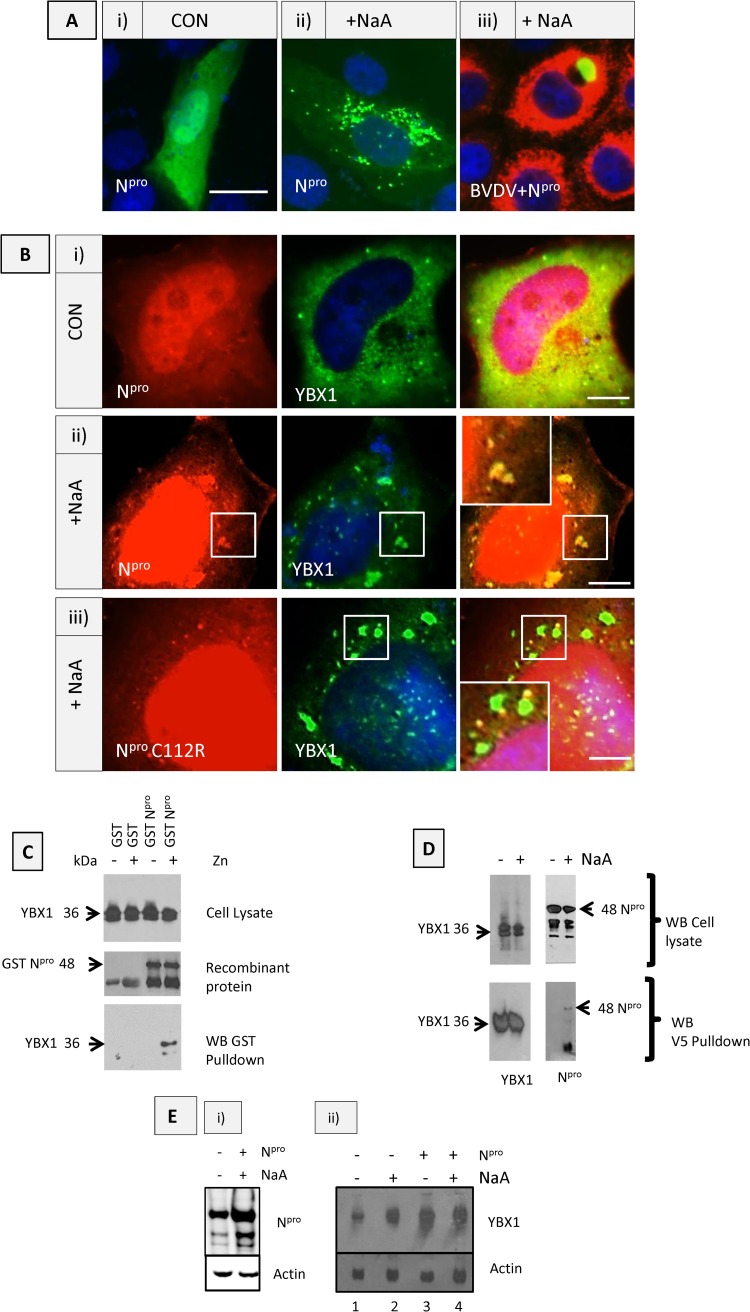
Interaction and colocalization of N^pro^ with Y-box protein 1 (YBX-1) requires the zinc-binding TRASH motif. (A) N^pro^ localization in BVDV-infected MDBK cells. (i) Control MDBK cells transfected with N^pro^-GFP. (ii) MDBK cells transfected with N^pro^-GFP and treated with NaA for 4 h. (iii) Cells infected with BVDV for 48 hpi, transfected with N^pro^-GFP for 24 h, and treated with NaA for 4 h. Virus was detected with V182 bovine hyperimmune serum and anti-bovine cy5 secondary antibody. (B) N^pro^ codistributes with YBX1 to large cytoplasmic stress granules. Cells stably expressing N^pro^-mCherry and transfected with YBX-1-V5 were either untreated or treated with NaA for 4 h. YBX1 was detected using anti-V5 MAb and anti-mouse Alexa 488 secondary antibody, and DNA was stained with 4′,6-diamidino-2-phenylindole (DAPI) (blue). (i) Control untreated HeLa cells, (ii) Cells stably expressing N^pro^-mCherry, transfected with YBX1-V5 and treated with NaA for 4 h. (iii) Cells stably expressing mutant N^pro^ C112R-mCherry and transfected with YBX1-V5 and treated with NaA for 4 h. (C) Protein pulldown of HeLa cell lysates with recombinant N^pro^ shows interaction with endogenous cellular YBX1 requiring zinc. The GST and GST-N^pro^ recombinant proteins were washed in zinc buffer or zinc chelator, TPEN. Glutathione-Sepharose beads were used to pull down GST-N^pro^ and its cobinding proteins. Top panel, endogenous YBX1 detected in cell lysates at 36 kDa with an anti-YBX1 antibody; middle panel, GST and GST-N^pro^ recombinant proteins detected using anti-GST antibody and anti-N^pro^, respectively; bottom panel, HeLa cell lysates incubated with GST and GST-N^pro^ proteins were pulled down with glutathione-Sepharose beads, washed extensively, and separated by SDS-PAGE. Proteins were transferred to membranes and probed with rabbit YBX1 antibody, which was detected with anti-rabbit-HRP by enhanced chemiluminescence (ECL). (D) Interaction of N^pro^ with YBX1 increases after stress. Cells stably expressing N^pro^-mCherry were transfected with YBX1-V5, and cells were untreated (−) or treated (+) with sodium arsenate for 4 h (NaA). Lysates were pulled down with anti-V5 antibody bound to Sepharose beads. Top panels, Western blots of cell lysates either untreated (−) or treated (+) with NaA to detect YBX-V5 with anti-V5 monoclonal antibody (left) or N^pro^ with rabbit anti-N^pro^ antibody (right) using anti-mouse or anti-rabbit HRP antibodies, respectively. Bottom panels, immunoprecipitation of proteins using an anti-V5 antibody bound to Sepharose beads, followed by transfer of proteins to membranes and Western blotting with anti-V5 (left panel) or anti-N^pro^ (right panel) antibodies. (E) N^pro^ and YBX accumulate following stress. (i) Lysates from control cells (−) or cells expressing N^pro^-mCherry (+ N^pro^), either untreated (−NaA) or stressed for 4 h (+NaA), were blotted with antibodies against N^pro^ and actin. (ii) Lysates from control cells (−) or cells expressing N^pro^ (+N^pro^) either untreated (−) or treated with NaA (+) for 4 h were blotted with anti-YBX1 and antiactin antibodies.

We have previously shown that ectopic expression of the single viral protein N^pro^ can inhibit apoptosis and interferon production through binding and degradation of IRF3 (8). Since we show here that N^pro^ is multifunctional and also binds to ribonucleoproteins, such as YBX1, a major component of stress granules, and that virus infection inhibits stress granule formation, we thought that Npro was a good candidate for the individual protein responsible for stress granule inhibition. Fluorescently tagged N^pro^ (N^pro^-GFP or N^pro^-mCherry) was expressed in cells. N^pro^ distributed to the nucleus and cytoplasm (Fig. 2Ai) and subsequently redistributed to cytoplasmic granules following stress with sodium arsenate (Fig. 2Aii). These cells were protected from apoptosis, as we have described in detail in our previous work (8), indicating stress granule assembly in the absence of apoptosis activation. However, when N^pro^-mCherry was expressed in BVDV-infected cells, N^pro^ relocated to an area associated with the viral replication complex (Fig. 2Aiii). In the next experiments, we focused on possible functional roles of N^pro^ by investigating its binding to, localization with, and effect on host proteins identified in Tables 1 and 2, outside the context of viral infection and in the absence of the other viral proteins. First, we investigated whether N^pro^ redistributed with YBX-1 in stressed cells. When coexpressed together, N^pro^ and YBX1 were both located to the cytoplasm in unstressed cells (Fig. 2Bi). Cells expressing N^pro^-mCherry and YBX1 treated with sodium arsenate for 4 h showed redistribution of N^pro^ to cytoplasmic granules containing YBX1 (Fig. 2Bii), indicating that expression of N^pro^ itself does not inhibit the formation of stress granule complexes in cells. In cells expressing the mutant N^pro^ C112R protein, perinuclear stress granules containing YBX1 were formed, but these granules no longer colocalized with N^pro^ C112R, which was found in smaller cytoplasmic foci (Fig. 2Biii, insert). Taken together, these results indicate that wild-type N^pro^ binds to YBX1 through its TRASH domain, since N^pro^ with the C112R mutation, which disrupts the zinc binding TRASH domain, no longer codistributes with YBX1 to stress granules. It demonstrates that N^pro^ relocation to the stress granule complex is attributable to its binding to host proteins, such as YBX1. During infection, however, we show that stress granule formation is inhibited and YBX1 remains cytoplasmic (Fig. 1A), whereas N^pro^ redistributes to the replication complex after infected cells are stressed (Fig. 2A). This suggests that since YBX1 is not recruited to the replication complex during infection, it is not required for viral replication, but it may have an important function bound to N^pro^ at sites of viral genome translation in the cytoplasm.

The binding of N^pro^ to YBX1 was confirmed biochemically by a coimmunoprecipitation assay using either recombinant N^pro^ GST (Fig. 2C) or lysates from HEK 293 cells expressing N^pro^-mCherry (Fig. 2D). Cell lysates were passed over glutathione beads bound to GST or N^pro^ GST that had been washed in zinc buffer or washed with a zinc chelator, TPEN (Fig. 2C). Proteins pulled down were separated by gel electrophoresis and blotted with an anti-YBX 1 antibody. YBX1 was pulled down with the N^pro^ recombinant protein specifically in the presence of zinc and not when the zinc had been chelated. This confirmed that N^pro^ interacts with YBX1 through its zinc-binding TRASH motif.

In the next experiment, cells stably expressing N^pro^-mCherry were cotransfected with a plasmid encoding YBX1 tagged with V5 to investigate binding in cells (Fig. 2D). Cells were treated with sodium arsenate for 4 h to promote the formation of stress granules. Proteins pulled down with YBX1 using anti-V5 antibody were separated on a gel and probed with anti-N^pro^ antibodies. N^pro^ was detected bound to YBX1 in cells that had been stressed but not in unstressed cells (Fig. 2D). This may reflect the increased affinity or abundance of the complex bound together in stress granules in cells.

In cells treated with sodium arsenate for 4 h, there was accumulation of the N^pro^ protein (Fig. 2Ei). Oxidative stress also increased the levels of YBX1 (Fig. 2Eii, lanes 1 and 2). In the presence of N^pro^, increased levels of YBX1 were precipitated both with and without arsenate treatment (Fig. 2Eii, lanes 3 and 4). These results show that both induction of stress and expression of N^pro^ led to the accumulation of YBX1. The requirement for YBX1 during BVDV infection was attempted by silencing of YBX1 in infected MDBK cells, but because of the extremely low rates of transfection of these cells and the high level of expression of YBX1, it was not possible. (Pestiviruses have a narrow tropism to their host species, and therefore gene knockdown experiments are not possible using knocked-out mouse embryonic fibroblasts [MEFs]).

### Colocalization of interacting host factors with N^pro^ after stress.

**FIG 3:**
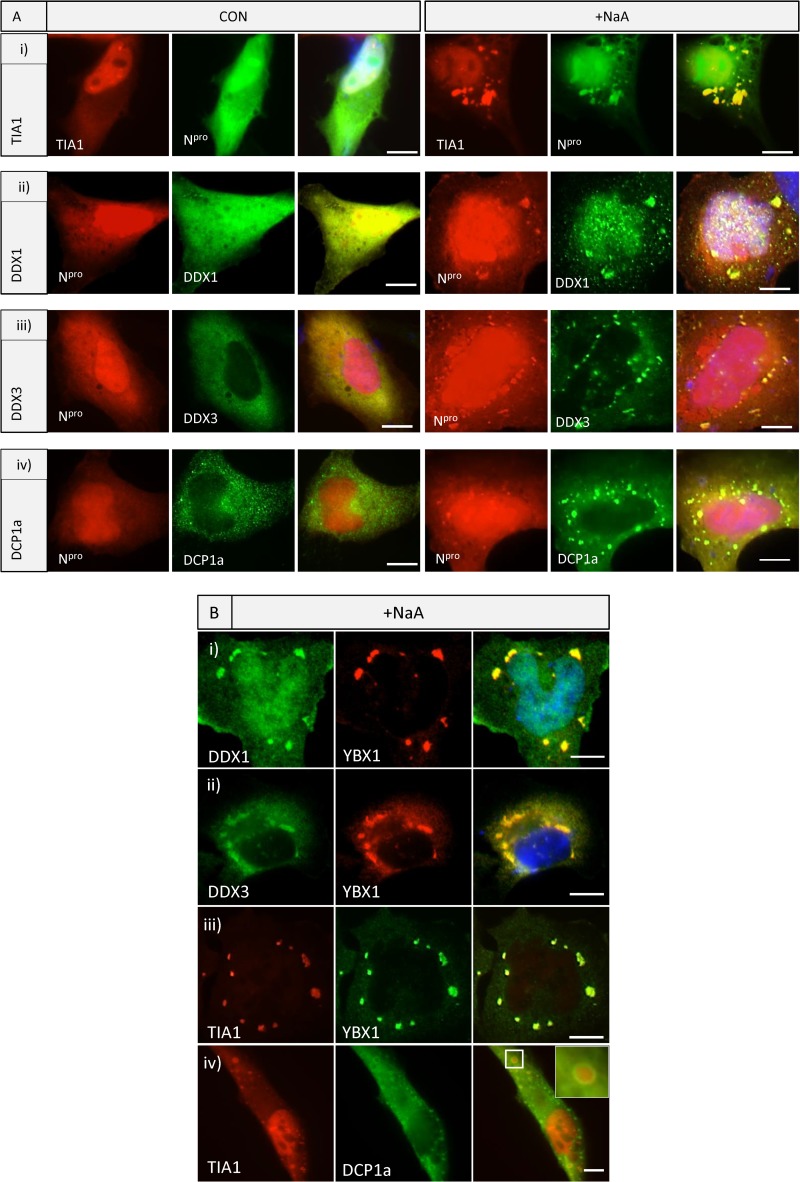
Pestivirus N^pro^ forms a complex with ribonucleoproteins and redistributes to stress granules. (A) N^pro^ redistribution with TIA1, DDX1, DDX3, and DCP1a following stress. Cells were either untreated (CON) or subjected to oxidative stress for 4 h (+NaA). Cell expressing N^pro^-GFP were cotransfected with TIA1-mCherry (i). Cells expressing N^pro^-mCherry were cotransfected with DDX1-V5 (ii), DDX3-Myc (iii), or DCP1a-V5 (iv), which were detected with anti-V5 or anti-Myc MAbs and secondary anti-mouse Alexa 488 antibodies. (B) Codistribution of YBX1 and TIA1-containing particles with RNA helicases DDX1 and DDX3. HeLa cells were transfected with plasmids encoding DDX1-V5 (i) or DDX3-Myc (ii) and treated with NaA for 4 h. Endogenous YBX1 was detected with rabbit anti-YBX1 and an anti-rabbit Alexa 594 secondary antibody, and DDX1 and DDX3 were detected with V5 and Myc MAbs, respectively, and an anti-mouse Alexa 488 secondary antibody. HeLa cells were cotransfected with a plasmid encoding TIA1-mCherry and YBX1-V5 (iii) or DCP1a-V5 (iv), detected using anti-V5 with anti-mouse Alexa 488 secondary antibody.

As an assay for N^pro^ interaction with factors identified by mass spectrometry, oxidative stress was used to determine their codistribution to granules. We coexpressed N^pro^-mCherry with TIA1, DDX1, DDX3, and DCP1a. Figure 3A shows codistribution of these proteins in the cytoplasm and their redistribution with N^pro^ to stress granules as an indication of cobinding. DDX1 was also found in smaller cytoplasmic foci. DDX1 and DDX3 are RNA helicases with roles in transcriptional and posttranscriptional RNA metabolism, including RNA splicing, translation, DNA repair, and transcription (10). The decapping enzyme DCP1a, a component of P bodies, which are related to stress granules but contain other proteins involved in mRNA decay (20), codistributed with N^pro^ to stress granules (Fig. 3Aiv). Stress granules are complex structures made up of RNA-binding proteins, 40S ribosomal subunits, but the composition can vary (20). We have shown that DDX1 and DDX3 aggregate into granules which also comprise YBX1 and TIA1 (Fig. 3Bi to iii), whereas DCP1a formed a ring-like structure around the TIA1-containing granules (Fig. 3Biv), demonstrating a separate domain for this protein. The imaging experiments, together with the proteomic data, show that N^pro^ interacts with important antiviral RNA binding proteins in the cytoplasm, required for translation. Pestivirus infection prevents stress granule aggregation by an unidentified mechanism, but expression of N^pro^ alone does not. During infection, the interaction may have an effect on viral or host translation.

### RNA helicase A and La autoantigen redistribute with N^pro^ from the nucleus to cytoplasmic stress granules.

**FIG 4:**
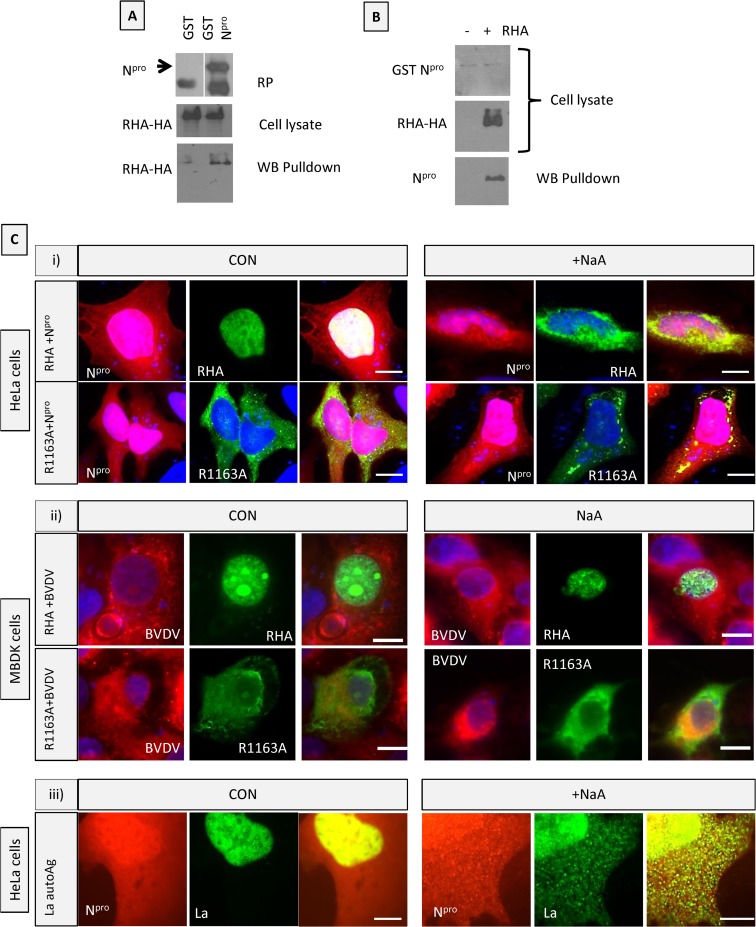
RNA helicase A (DHX9) binds to N^pro^ and redistributes from the nucleus to cytoplasmic stress granules following stress. (A) RHA tagged with HA was transfected into HEK293 cells, and lysates were incubated with GST or GST-N^pro^ recombinant proteins. Cobinding proteins were pulled down with glutathione-Sepharose beads. Top panel, Western blot of recombinant protein with anti-GST antibody (RP); middle panel, Western blot of cell lysates expressing RHA with anti-HA antibody detected with anti-mouse HRP secondary antibody; bottom panel, Western blot of proteins pulled down with glutathione-Sepharose beads and detected with anti-HA antibody. (B) GST-N^pro^ expressed alone (−) or with RHA-HA (+) in HEK293 cells was pulled down using glutathione-Sepharose beads from cells lysates. Top panel, Western blot of cell lysates with anti-N^pro^ antibody; middle panel, Western blot of cell lysates with anti-HA antibody; bottom panel, Western blot of proteins pulled down with glutathione beads using anti-N^pro^ antibody. (C) RNA helicase A (DHX9) redistributes from the nucleus to cytoplasmic stress granules with N^pro^ following stress. (i) HeLa cells were cotransfected with N^pro^-mCherry and either RHA-GFP (RHA +N^pro^) or mutant RHA R1163A-GFP (R1163A +N^pro^). Cells were either untreated (CON) or subjected to oxidative stress (+NaA). (ii) MBDK cells were infected with BVDV for 48 h and transfected with either RHA-GFP or mutant RHA R1163A GFP for 24 h. Cells were either untreated (CON) or subjected to oxidative stress (+NaA). (iii) HeLa cells were cotransfected with N^pro^-mCherry and La autoantigen-GFP. Cells were either untreated (CON) or subjected to oxidative stress (+NaA).

Another important protein shown to interact with N^pro^ is RNA helicase A (RHA). RHA (also known as DEAH box protein 9 [DHX9]) binds dsRNA; it is a DExD/H box helicase with both DNA and RNA helicase activity. Its antiviral properties include binding to the RNA-induced silencing complex (RISC) to help load dsRNA onto Argonaute for microRNA (miRNA) production (37). To confirm interaction between RHA and N^pro^, cells were transfected with a plasmid encoding RHA tagged to a hemagglutinin (HA) epitope. Cell lysates were incubated with beads bound to GST or GST-N^pro^ recombinant protein and washed as described. RHA was pulled down with beads containing GST N^pro^ but not GST beads alone (Fig. 4A), showing specific binding of RHA by recombinant N^pro^. When HEK 293 cells stably expressing cDNA for GST N^pro^ were cotransfected with a plasmid encoding RHA-HA, GST-N^pro^ was pulled down by GST in cells overexpressing RHA but not in cells without RHA (Fig. 4B). This indicates an increased efficiency of GST binding and N^pro^ pulldown in the presence of RHA, possibly due to conformational change. Next we investigated the localization of RHA in cells expressing N^pro^. HeLa cells were cotransfected with RHA-GFP and N^pro^-mCherry. In untreated cells, RHA localized to the nucleus (Fig. 4Ci, top left panels), and there was little staining in the cytoplasm, whereas N^pro^ expression was both nuclear and cytoplasmic. When these cells were subjected to oxidative stress, RHA relocated from the nucleus to cytoplasmic stress granules (Fig. 4Ci, top right panels, +NaA), and these granules colocalized with N^pro^. Since pestiviruses replicate in the cytoplasm with no nuclear component, we used RHA-GFP carrying a mutation (R1163A) which blocks its nuclear translocation (25) to investigate the effect on RHA, which is expressed exclusively in the cytoplasm. In unstressed cells, the mutant RHA-GFP R1163A was seen only in the cytoplasm (Fig. 4Ci, bottom panels). Treatment with sodium arsenate led to large cytoplasmic stress granules that colocalized with N^pro^-mCherry, clearly indicating RHA binding in a complex to N^pro^ in cytoplasmic stress granules (Fig. 4Ci, bottom panels).

In order to investigate RHA relocalization in BVDV-infected cells, MDBK cells were infected with virus and transfected with RHA-GFP (Fig. 4Cii). In control BVDV-infected cells, RHA-GFP distributed to the nucleus. When cells were stressed, there was no change in the distribution of RHA-GFP, which remained in the nucleus (Fig. 4Cii, top right panel.) This confirms the results shown in Fig. 1, that stress granules are not formed when BVDV-infected cells are stressed and RHA, TIA1, and YBX1 do not redistribute into stress granules. Similarly, the RHA R1163A -GFP mutant remained cytoplasmic both before and after treatment with sodium arsenate and did not relocate to stress granules (Fig. 4Cii, lower panels).

We have shown by mass spectrometry that La autoantigen interacted with N^pro^ (Table 1). La autoantigen is an RNA-binding protein which binds several viral RNAs, including those of poliovirus and human immunodeficiency virus (HIV), to enhance their translation *in vitro* (38). In the next experiment, we showed that the La autoantigen also redistributes from the nucleus to cytoplasmic granules with N^pro^ following induction of stress (Fig. 4Ciii). La-GFP was predominantly nuclear before stress, but after treatment it was seen in small cytoplasmic dots colocalizing with N^pro^ (Fig. 4Ciii). Thus, we have identified components of ribonucleoparticles made up from both cytoplasmic and nuclear proteins that bind to N^pro^ in cells and which are redistributed to a cytoplasmic complex together after stress.

### Pestivirus N^pro^ does not suppress RNA interference in mammalian cells.

**FIG 5:**
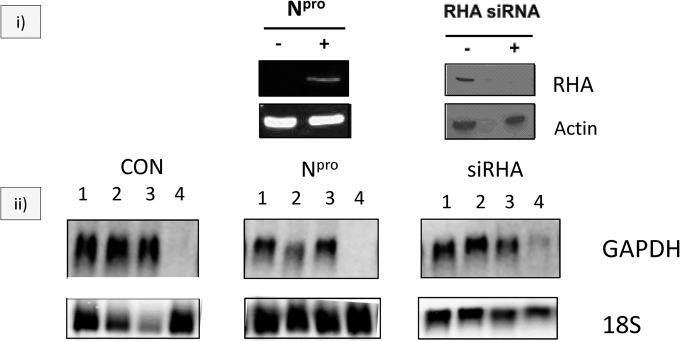
N^pro^ does not suppress RNA silencing.(i) Western blot of control HeLa cells (−), cells stably expressing N^pro^-mCherry (+N^pro^) (left panel), or HeLa cells silenced for RHA expression (RHA siRNA) (right panel). N^pro^ was detected with a rabbit anti-N^pro^ antibody and visualized with IRDye-labeled secondary antibodies. RHA was detected with mouse anti-RHA and visualized with HRP-labeled secondary antibody. (ii) Northern blot analysis of GAPDH mRNA levels following GAPDH silencing using a ^32^P-labeled GAPDH-specific primer. 1, control cells; 2, mock-treated cells; 3, nontargeting siRNA-treated cells; 4, GAPDH siRNA-treated cells. Control cells (left panel), cells expressing N^pro^ (middle panel), and cells silenced for RHA (right panel) are shown. An 18S RNA blot is shown for equal loading.

RNA granules aggregating with N^pro^ are involved in several different RNA metabolic processes, and some of the components, for example RNA helicase A, interact directly with the RNA interference (RNAi) pathway (37). Silencing of RHA has been shown to block the RNAi pathway, and RHA is important for loading short RNAs, including siRNAs and miRNAs, onto RISC (37). The interacting protein La autoantigen is an RNAi activator that promotes the release of cleaved mRNA from Ago2 and efficient RNAi, an antiviral defense (39). Since N^pro^ is a viral suppressor of interferon synthesis and apoptosis, and other viral suppressors of interferon, such as Ebola vp35, vaccinia virus E3L, and influenza virus NS1, also block the RNAi pathway, we investigated whether N^pro^ was a viral suppressor of RNA silencing (22, 23). In the next series of experiments, the GAPDH housekeeping gene was silenced using GAPDH-specific siRNAs in control or N^pro^-expressing cells or cells where RNA helicase A had been silenced (RHA siRNA). GAPDH RNA levels were analyzed by Northern blotting with a ^32^P-labeled GAPDH-specific primer (Fig. 5ii). GAPDH was efficiently knocked down by siRNAs in control cells (Fig. 5ii, left panel, lane 4). There was no difference in GAPDH knockdown between control and N^pro^ mCherry-expressing cells (Fig. 5ii, middle panel, lane 4), showing that N^pro^ does not suppress RNAi from exogenously added siRNAs. It has been previously reported that RNA helicase A inhibits RISC loading (37) and that it might be involved in RNAi, so we investigated whether silencing of RHA had an effect on the RNAi assay of GAPDH knockdown with GAPDH-specific siRNAs, as described above. We silenced RHA with RHA-specific siRNAs (Fig. 5i, right panel). RHA depletion shows some suppression of RNAi from externally added siRNAs (Fig. 5ii, right panel, lane 4), as has been reported previously (37).

## DISCUSSION

Pestiviruses are cytoplasmic RNA viruses that must evade host innate immune responses, which include stress, apoptosis, and interferon induction. In this study, we have shown that BVDV can inhibit stress granule formation, adding this to the list of evasion strategies of this virus (7, 8, 40). Viruses must prevent translational silencing in their host cell in order to survive. Many RNA viruses inhibit stress granule formation during infection, whereas others induce them to then inhibit at later times in infection (41). In some cases, virus infection itself can trigger stress granules as an antiviral pathway through the activation of kinases, such as protein kinase R (PKR), that phosphorylate eIF2 to shut off host translation and limit viral replication (42). In the absence of stress and other viral proteins, our proteomics analysis found that N^pro^ bound to many RNA binding proteins, such as the 40S and 60S ribosomal proteins, ribonucleoproteins, and numerous other components of translational machinery in the cytoplasm, including YBX-1, RHA (DHX9), IGF2BP1, DEAD box helicases, eIF3, PABP-1, and heterogeneous nuclear ribonucleoproteins (hnRNPs) (30). We found that the binding was not dependent on the presence of viral RNA or dsRNA [poly(I·C)]. In BVDV-infected cells which had been stressed, N^pro^ redistributed from the cytoplasm to the replication complex, whereas RNP proteins remained cytoplasmic and stress granules were not seen. In uninfected cells, N^pro^ bound to ribonucleoproteins and ribosomal proteins in the cytoplasm, and it was assembled by them into stress granule complexes, since N^pro^ mutated in the C-terminal TRASH motif was no longer assembled into stress granules. Therefore, it is unlikely that N^pro^ is the viral protein responsible for preventing the redistribution of RNPs to stress granules in BVDV-infected cells. This is in contrast to picornaviruses, where expression of the leader (L) protein was sufficient to inhibit stress granule assembly (43). It is possible that N^pro^ recruits these proteins at some point during infection for viral RNA translation. Another possible effect is to stall the translation of host proteins, but only at early stages of infection, since cells persistently infected with pestiviruses do not show translation arrest, and cells are protected from apoptosis (38). We describe here the proteomic content of the RNPs to which N^pro^ binds, but how the proteomic composition is linked to function or changes in different cellular compartments is largely unknown (30). Some RNA viral proteases, such as poliovirus proteases, actively bind and cleave stress granule proteins, including G3BP, PABP, and eIF4G, early during infection (44); nevertheless, stable stress granules containing TIA1 and positive-sense mRNAs form, promoting host cell shutoff (45). The pro- or antiviral functions of stress granule proteins are still unclear, since they act as sites of viral and mRNA sequestration and translational initiation and repression. They may act to reprogram protein expression by silencing mRNAs selectively during infection and also to sequester viral transcripts.

We have shown that N^pro^ interacted with YBX1. Interestingly, this is a novel partner of NS3 in hepatitis C virus infection, which redistributes to lipid droplets for virus assembly to control the balance between infectious particle production and viral RNA replication (46). In alphavirus infections, the Sindbis virus protein nsP3 is also found in YBX1 protein-RNA complexes in endosome-like vesicular organelles (47), where they have a function in viral RNA synthesis. In addition, N^pro^ interacts with many of the same proteins seen to interact with the YBX1 interactome, including IGFBP2, DDX3, ILF2, and RHA (DXH9). These are multifunctional proteins, and it has been suggested that the YBX1 macromolecular complex has a panviral role in modulating virus production (48).

Both interacting partners RHA and La autoantigen are part of ribonucleoprotein particles, and we have shown here that they can be transported rapidly from the nucleus to the cytoplasm following stress. Nuclear pre-mRNA is packaged into hnRNPs and transported out of the nucleus. Several hnRNP proteins were identified as binding to N^pro^ in our mass spectrometry analysis. During infection, RHA may be recruited to viral RNA (vRNA), since other studies have shown RHA binding to 5′ and 3′ untranslated regions (UTRs) of pestivirus RNA to increase replication, which would indicate a proviral effect (49), whereas binding to the 3′ untranscribed region (UTR) of Dengue virus mediates an antiviral effect (50). The cellular reorganization of RHA with N^pro^ seen in this study is similar to that seen during picornavirus infection, where RHA plays an important part in its replication (51). RHA is phosphorylated by dsRNA binding kinase PKR, normally activated upon viral infection (52), and also binds to nuclear factor kappa B p65, a central regulator of the inflammatory response (53). Other N^pro^-interacting proteins may have proviral roles; for example, DDX3 binding to vaccinia virus K7 (24) and hepatitis C virus (HCV) core promotes replication (54). Interesting corroboration of the interactions described in this paper comes from a broad study using 70 viral immune modulators from 30 viral species, where they found many of the same interactors we show in Tables 1 and 2 (55). This shows that many other viruses also target this cellular pathway.

The localization of RNAi machinery in stress granules highlights their antiviral role in targeting viral RNA, and viruses must subvert RNAi activity, which would otherwise degrade their genomes. Another important protein found to interact with N^pro^ is poly(ADP-ribose) (PARP), which modifies Argonaute 2 function and has a role in miRNA generation (56). Our experiments here show that N^pro^ expression does not suppress the exogenous siRNA pathway, although it is possible that binding of N^pro^ to PARP may repress miRNA production. The host factors RHA and La autoantigen are also important in RNA interference, being involved in RISC loading and unloading (37, 38). We demonstrated that RHA binds to N^pro^ in stress granules, and we asked if N^pro^ inhibited RNAi by using siRNAs to silence the GAPDH housekeeping gene. N^pro^ did not inhibit exogenous siRNA silencing of GAPDH. These results suggest that N^pro^ is not a viral suppressor of silencing (VSS) in mammalian cells or at least is not involved in the siRNA pathway. In some systems, viral antagonists of interferon have been shown to inhibit RNAi but not in some mammalian cells (57).

In summary, the N-terminal protease, N^pro^, of pestiviruses forms a complex with ribosomal and ribonucleoproteins in the cytoplasm which serves to recuit the machinery for translation and production of viral particles. Pestiviruses themselves inhibit oxidative stress-induced stress granule formation, which would prevent inhibition of cellular protein synthesis and promote viral protein synthesis. The modulation of viral replication by these interacting protein remains to be determined.
